# Clinical oncology in Malaysia: 1914 to present

**DOI:** 10.2349/biij.2.1.e18

**Published:** 2006-01-01

**Authors:** GCC Lim

**Affiliations:** Department of Radiotherapy and Oncology, Kuala Lumpur Hospital, Kuala Lumpur, Malaysia

**Keywords:** History, cancer, radiotherapy, oncology, Malaysia

## Abstract

A narration of the development of staff, infrastructure and buildings in the various parts of the country is given in this paper. The role of universities and other institutions of learning, public health, palliative care, nuclear medicine and cancer registries is described together with the networking that has been developed between the government, non-governmental organisations and private hospitals. The training of skilled manpower and the commencement of the Master of Clinical Oncology in the University of Malaya is highlighted. Efforts taken to improve the various aspects of cancer control which includes prevention of cancer, early detection, treatment and palliative care are covered. It is vital to ensure that cancer care services must be accessible and affordable throughout the entire health system, from the primary care level up to the centres for tertiary care, throughout the whole country.

## INTRODUCTION

Cancer is an increasing health problem in Malaysia. Good and comprehensive cancer treatment is the right of all. The development of the discipline of Clinical Oncology, previously known as Radiotherapy and Oncology, is described in this paper. Much has been achieved in clinical oncology in the past 90 years in Malaysia.

## KUALA LUMPUR

The earliest records of x-ray therapy in Malaysia show the acquisition of a Crookes x-ray tube in Singapore in 1914 and a Coolidge tube in 1920 [1]. Radium sources were used in the treatment of gynaecological malignancies, skin lesions and implants of accessible lesions. The radium needles and radium tubes from the General Hospitals of Ipoh, Johor Bahru and Penang were later transferred and stored in the Department of Radiotherapy and Oncology at Hospital Kuala Lumpur [2]. A 140 kV deep x-ray therapy unit was installed in Singapore General Hospital in 1933.

In the 1950's, external beam radiotherapy was delivered in Kuala Lumpur with the Philips Dermopan 50 kV superficial x-ray machine (Philips NV, Eindhoven, Netherlands), 200 kV Muller x-ray machine and 250 kV rotating Muller x-ray machine [1].

**Figure 1:**
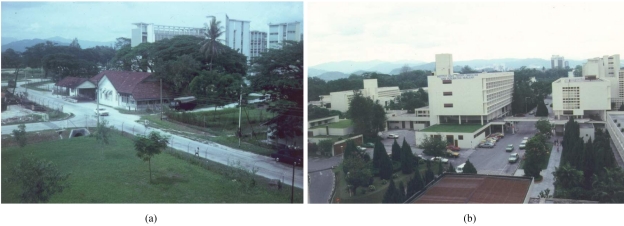
The Department of Radiotherapy and Oncology in the (a) 1950's and (b) 1968

The department was housed in a wooden building between the Maternity Hospital and the Neurosurgical unit (Figure 1a). There were no dedicated beds in the unit at that time [3]. From 1950 to 1960, radiotherapy services were provided by Dr Lynch, an Irish radiotherapist who shuttled between Singapore and Kuala Lumpur, and Dr Lal from Singapore [4]. The support staff comprised two radiographers, a staff nurse, a secretary/receptionist and an attendant [2].

**Figure 2:**
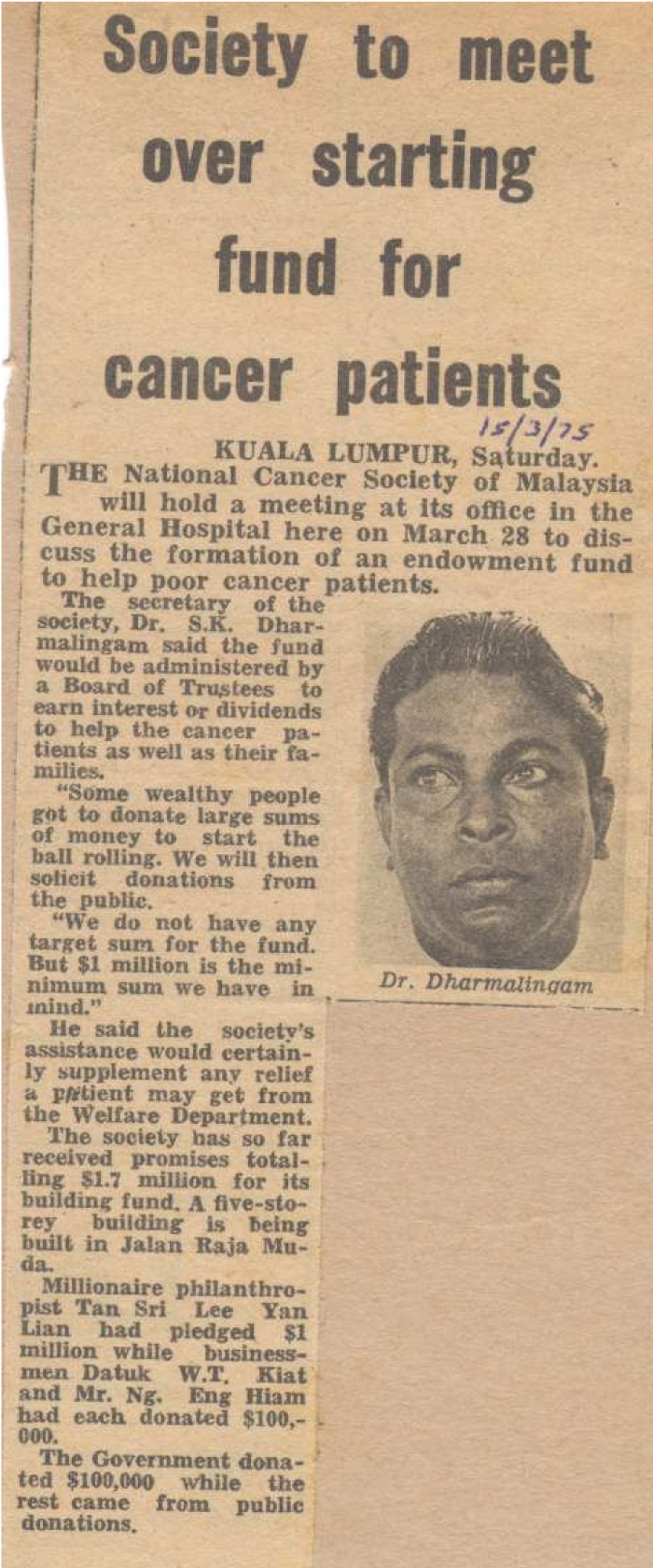
Dato' Dr. S.K. Dharmalingam was the driving force in the early days of Radiotherapy and Oncology in Malaysia.

Dato' Dr. S.K. Dharmalingam (Figure 2) succeeded Dr. Lynch after returning from the Middlesex Hospital in the United Kingdom in 1960 and was appointed as the first Malaysian Consultant Radiotherapist. Two more radiographers and a physicist were then recruited. The staff comprised 4 local consultants, 8 sisters, 28 staff nurses, 40 assistant nurses and approximately 70 attendants. Four student radiographers were initially trained in Kuala Lumpur for one year followed by further training in Hong Kong, while two staff nurses were sent for training in the United Kingdom. Overseas specialists attached to the department included Professor Roberts of Middlesex Hospital, Professor K. Brittan from St Bartholomew's Hospital, as well as doctors from Korea, Pakistan and Austria [3].

Additional equipment that was acquired in 1965 included a radium safe costing $35,000 and a 300 kV orthovoltage treatment machine. When Tun Omar Ong Yoke Lin and Tun Tan Siew Sin were the Minister of Health and Minister of Finance respectively, an allocation of MYR 3 million was made for the establishment of the new Institute of Radiotherapy, Oncology and Nuclear Medicine at the Kuala Lumpur General Hospital. The physical construction of the building was started in August 1967 and was completed in November 1968 (Figure 1b). The facility had 180 beds, its own operation theatre for brachytherapy, a separate laboratory, a pharmacy, and an outpatient department. The Cancer Ambulant Ward was established to cater to cancer patients from other states in the country who needed accommodation but were well enough not to be in a regular ward. Before, such patients had been put up in the New Hotel, through the National Cancer Society of Malaysia, and at the old Tai Wah Hospital, which was refurbished [3]. The equipment included a Siemens Betatron (Siemens AG, Erlangen, Germany) that provided electron treatments ranging from 5 MeV to 43 MeV, and two single energy 6 MV linear accelerators (MEL-75) [1].

Nuclear medicine was started under the Institute of Radiotherapy, Oncology and Nuclear Medicine in 1962 [2]. The first technician, Mr. Anthony Ng was sent to the Royal Melbourne Institute of Technologists for training [3]. The Radioisotope Laboratory was established at the University Hospital, Kuala Lumpur in 1967 [1].

In 1974, a physicist post was created in the engineering division in the Ministry of Health. A physics workshop with two lathe machines, a shaping machine, drilling, cutting machines and a physics mould room were also installed in the institute for preparation of lead shielding devices and other special treatment devices. Two Farmer Dosemeters type 2502 (PTW-Freiburg, Freiburg, Germany) were used for dose calibration and measurement of the megavoltage machines. The analog Farmer Dosemeters have been replaced by digital dosemeters for more accurate dosimetry. A single energy linear accelerator and a telecaesium unit were installed in 1977 and 1978 respectively to replace the orthovoltage machines. The physicists were doing the isodose planning by manual summation until a computerised treatment planner, Mevaplan (Siemens AG, Erlangen, Germany), was introduced under the project of upgrading and replacement of old machines in 1987 and 1988 [5]. Shielding of critical structures was based on check films done with lead cut-outs and lead blocks [6]. Radium-226 was the earliest radioisotope used in brachytherapy. Caesium-137 replaced radium-226 from 1993 until 2004, after which iridium-192 was used.

**Figure 3:**
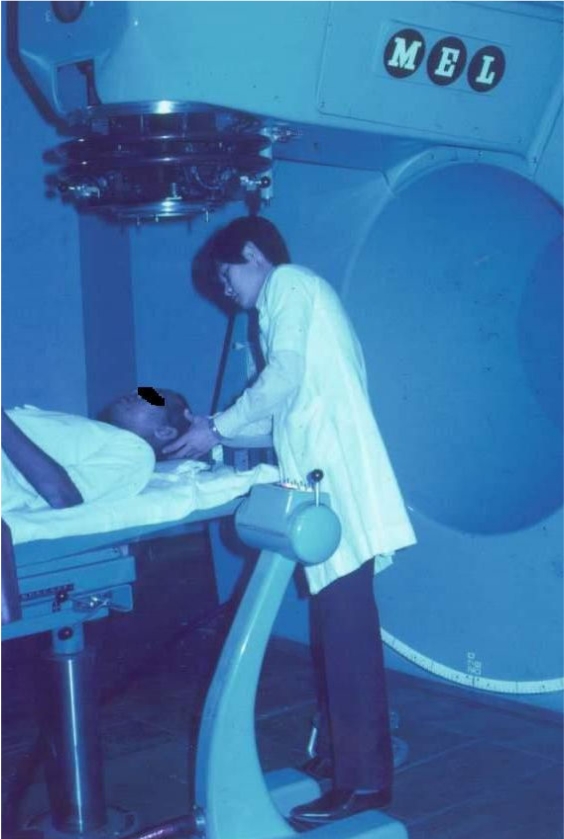
Mr. F.C. Fam with a patient on one of the earliest linear accelerators in the department.

**Figure 4:**
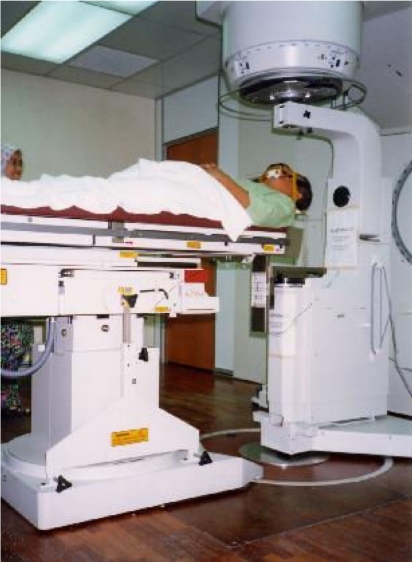
A patient undergoing total body irradiation.

**Figure 5:**
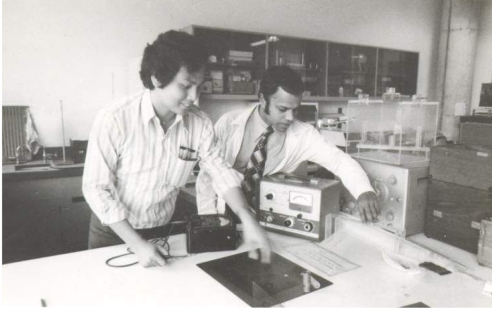
Mr. L. Lee and Mr. T. Yogaratnam in the Physics Laboratory.

**Figure 6:**
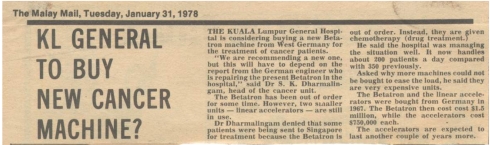
The state of the treatment machines in 1978.

Phase II development of the Institute which was completed in 1995 established facilities such as Day Care and saw the renovation of the operation theatre. Equipment that were installed since then included two new linear accelerators in 1997 and 2001, a high dose rate remote afterloading brachytherapy system in 1997, three-dimensional treatment planning system and a digital imaging simulator in 2001. Services such as total body irradiation benefited patients requiring bone marrow transplant, not only at the Hospital Kuala Lumpur, but also at the University of Malaya Medical Centre as well. Some of the early treatment machines and personnel are shown in Figures 3 to 6.

Stereotactic radiosurgery began in Hospital Kuala Lumpur on 4 March 1999. High dose rate remote afterloading system for brachytherapy has completely replaced the manual afterloading system since October 2003 in Hospital Kuala Lumpur.

## PENANG

The service in Penang was upgraded in 1996 with the posting of Dr. D. Jayendran, a senior clinical oncologist from the Ministry of Health. Chemotherapy and palliative care was provided at Penang Hospital while radiotherapy was provided for government patients at Mount Miriam Hospital and Pantai Mutiara Medical Centre.

## EAST MALAYSIA

The Department of Radiotherapy and Oncology in Sarawak General Hospital was officially opened on 15 August 1985. The first clinical oncologist to serve here was Dr. J. Singh. The Nuclear Medicine section was started soon after. In 1993, day chemotherapy was started in the department. Currently, the department has 68 beds. It originally had two linear accelerators, a conventional simulator and a Buchler medium dose rate remote after-loading brachytherapy machine (Amersham plc, Buckinghamshire, United Kingdom) that used caesium-137 as the radioactive source. A new cobalt unit was installed in the department in 1996. One of the linear accelerators was replaced with a new one in 1997. In June 2001, the Buchler brachytherapy unit was replaced with a high dose rate brachytherapy unit. Patients with nasopharyngeal cancer could now be treated with brachytherapy in Sarawak General Hospital. Previously, the brachytherapy machine only treated patients with gynaecological malignancies [7].

**Figure 7:**
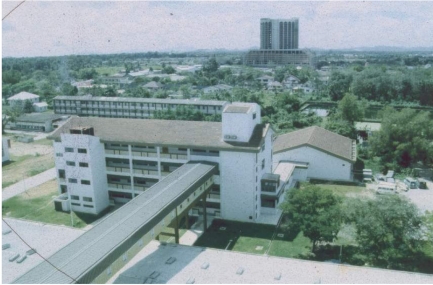
The Radiotherapy Unit is connected to the main building of the Sarawak General Hospital by a covered walkway.

Palliative care service was started in the Department of Radiotherapy and Oncology and Hospice home care service was started in 1995 in Kuching. From 1996 onwards, the Hospice home care service was extended statewide after more nurses were trained from various parts of Sarawak. In 2003, a dedicated Palliative Care Ward was completed. The department now has 16 beds for palliative care, with 8 beds for acute care and 8 beds for continuing care. The Palliative Care Ward has the task of ensuring that palliative care service is developed properly and that the service is available to patients in Sarawak. A system of drug delivery was also set up to ensure that patients even in the most remote parts of Sarawak get the drugs for symptom relief. Palliative care training for doctors, nurses and paramedics has been conducted by the Department of Radiotherapy and Oncology in Sarawak General Hospital since 1995 (Figure 7). The training sessions were initially conducted in Sarawak General Hospital, and extended to Sibu in 1996 and Miri in 1997.

Early Cancer Surveillance Training was started in 1995 as more than 70% of patients with common cancers, that is, nasopharyngeal, breast and cervix, were in an advanced stage. Training, which involved theory as well as practical sessions, was given to nurses and medical assistants from various hospitals and peripheral clinics.

The Breast Support Group was started in 1993. In addition to its regular monthly Breast Support Group meeting, the group has hosted annual dinners and regular parties for patients in the department. Separate Cancer Patient Support groups for male and female patients, as per their request, was started in 1997. The patients meet regularly to discuss and share matters of common interest.

Dr. P. Pisani from the Epidemiology Unit of the World Health Organisation (WHO), Lyon, France visited Sarawak from 6-10 of June 2001 to assist Dr. B. Devi on data analysis on the incidence of nasopharyngeal cancer in Sarawak [8].

While there was a government facility in Sarawak, the services were provided by Sabah Medical Centre, a private facility from which the government purchases radiotherapy services.

## UNIVERSITIES

The academic unit was started in the University Hospital Kuala Lumpur (currently known as the University of Malaya Medical Centre) in 1997, with state-of-the-art equipment, including linear accelerator with multileaf collimators, stereotactic radiotherapy, high dose rate and low dose rate remote afterloading brachytherapy, and virtual simulation. The oncologists were Dr. I. Wahid, Dr. M.M. Abdullah; physicists were Ms. N. Whylde, Mr. B.H. Khoo, Ms. P. Rassiah; and the therapy radiographer was Mr. T. Yogaratnam.

Other universities with radiotherapy and oncology departments were Hospital Universiti Kebangsaan Malaysia and Hospital Universiti Sains Malaysia in Kubang Kerian, Kelantan.

The Department of Nuclear Medicine, Radiotherapy and Oncology of the Universiti Sains Malaysia, Kubang Kerian Campus started the first Radiotherapy service in the east coast of Peninsular Malaysia. The Radiotherapy and Oncology division was added to the existing Nuclear Medicine department in December 1995. The Radiotherapy and Oncology service was started with the guidance of Professor M. Embong (then Dean of Medical School) and Assoc. Professor A. Zakaria (Medical Physics). Ministry of Health seconded a clinical oncologist, Dr. D. Jayendran, and two therapy radiographers, Ms. A. Shaari and Mr. K. Hassan, to the Ministry of Education for this purpose. In 1999, the high dose rate brachytherapy service was operational with the collaboration of IAEA (International Atomic Energy Agency) and now this department is one of the departments offering high dose rate interstitial brachytherapy services in Malaysia. Subsequently, state-of-the-art radiotherapy techniques like three-dimensional conformal radiotherapy and x-knife radiosurgery service were added in 2001 [9].

## OVERVIEW OF RADIOTHERAPY SERVICES IN MALAYSIA

Today there are 19 centres for Radiotherapy and Oncology in Malaysia, comprising 5 government centres and 14 private centres. There are 25 linear accelerators, 7 cobalt-60 teletherapy machines, 15 brachytherapy units, 11 simulators and 4 computerised tomography-simulation units.

Forty-one hospitals in the Ministry of Health are providing chemotherapy services in addition to universities and the private sector. Conventional chemotherapy is given in many general hospitals, district hospitals and private centres. High dose chemotherapy with bone marrow rescue is provided by paediatric oncologists and haematologists in University Hospital Kuala Lumpur and Hospital Kuala Lumpur.

In order to decrease the burden on government centres, the government has purchased private radiotherapy services from Mount Miriam Hospital, Pantai Mutiara Medical Centre, Mahkota Medical Centre, Pantai Ayer Keroh Medical Centre, Nilai Cancer Institute, Gleneagles Oncology Centre and Sabah Medical Centre.

Quality assurance is an essential part of radiotherapy services and much work has been put into it since the early days. Quality assurance ensures both effectiveness and safety of radiotherapy services [22].

## THE BURDEN OF CANCER

**Figure 8:**
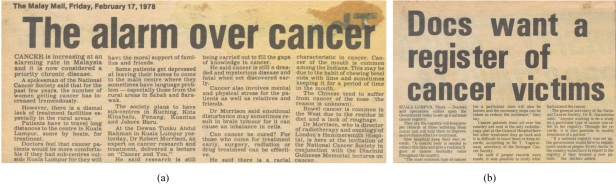
(a) The alarm over the incidence of cancer was felt in this country in 1970's; (b) The need for a national cancer registry was expressed a quarter of a century ago.

**Figure 9:**
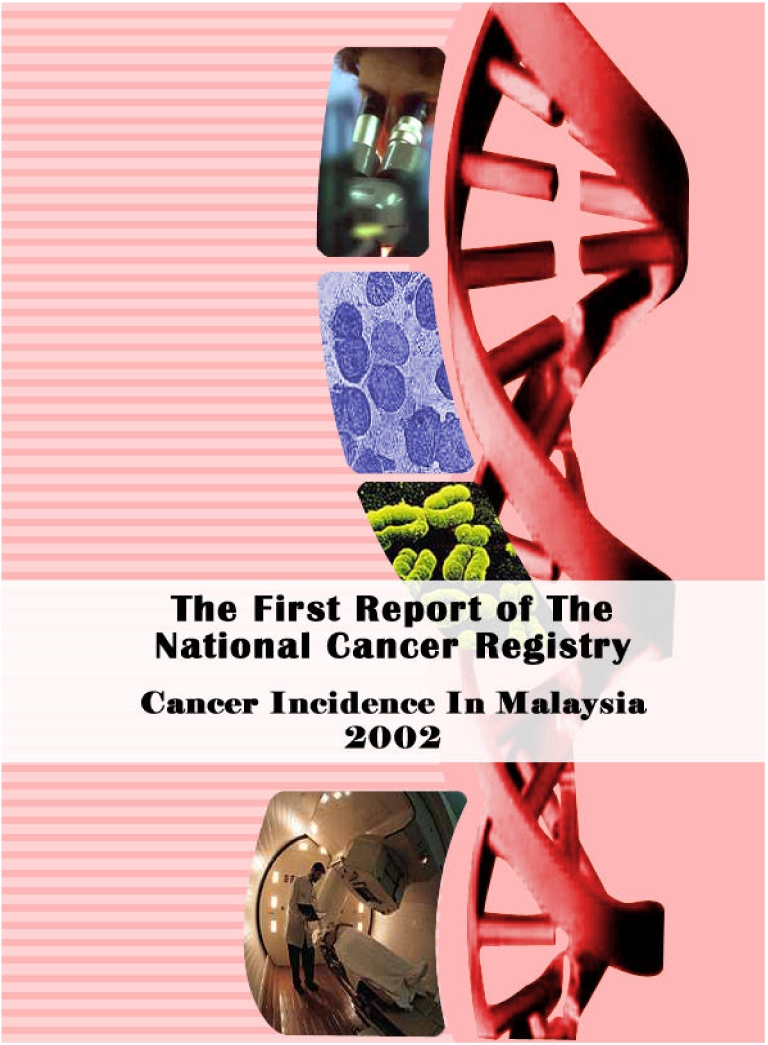
The First Report of the National Cancer Registry.

While the idea of a National Cancer Registry was first reported in the Star on 7 April 1978, the First Report of the National Cancer Registry was only realised on 4 July 2003 (Figures 8 and 9). For the first time, the real cancer burden in Malaysia was confidently estimated. A total of 26,098 patients were diagnosed with cancer among all residents in Peninsular Malaysia in 2002. The corresponding figures for Sabah and Sarawak were 1,748 and 2,002, respectively. The cumulative risk of Malaysians getting cancer in their lifetime was 1 in 5. Taking into account the unregistered cases, the cumulative lifetime risk was 1 in 4 [10]. The Penang Cancer Registry in its Five Year Report from 1994-1998, published on 15 December 2003 [11], demonstrated that 53.1% of cancers were in advanced stages, that is, Stage III or Stage IV.

**Figure 11:**
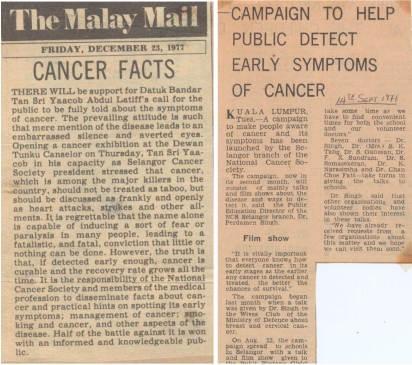
Public education on cancer.

**Figure 12:**
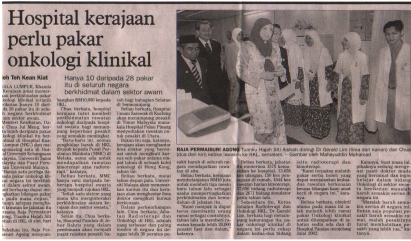
The visit by HRH Raja Permaisuri Agong on 7 June, 2001.

The role of public health cannot be overestimated. Public education, awareness campaigns and the promotion of healthy lifestyles must be emphasised while not neglecting the development of facilities for treatment. Efforts have been made to enhance prevention of cancer, screening, early detection of disease, equitable and accessible treatment, rehabilitation and palliative care (Figures 11 and 12). The Ministry of Health launched its campaign against cancer as a part of its ongoing Healthy Life Style approach to the prevention and control of some of our major public health problems in 1995 [12].

Networking between the public and private sectors and non-governmental organisations, establishment and upgrading of treatment facilities, and training of skilled staff in the treatment of cancer have been highlighted [13].

Decreasing the proportion of cancers presenting at a late stage would lead to increasing the chances of cure. In Sarawak, where the three commonest cancers are present at an advanced stage in at least 70% of the cases [14], efforts have been made at early detection. Allied health professionals, such as medical assistants and nurses have been trained to detect early signs and symptoms of cancers of the nasopharynx, breast and cervix.

Despite the awareness campaigns and public talks, the practice of screening for cancer is not widespread enough. The Second National Health and Morbidity Survey [15] revealed that the overall prevalence of Pap smear was only 26%, while the overall prevalence for breast self examination was 46.8%. Screening rate by Breast Self Examination was 34.1%, followed by Health Worker Examination (31.1%). Mammography was carried out only in 3.8% of women. These figures underscore the need for health education programmes to target population subgroups that would benefit from screening.

## PALLIATIVE CARE

Efforts at improving palliative care services throughout the country are being actively undertaken by governmental and non-governmental agencies.

Palliative Care was introduced by non-governmental organisations (NGOs) in Kuala Lumpur and Penang in 1991, by the Ministry of Health in Sabah in 1995. Among the first non-governmental organisations involved in palliative care was Hospis Malaysia. In 2000 a National policy was launched. Palliative Care continues to be NGO-driven. Most training programmes in Palliative Care are still run by NGOs. The NGOs continue to work closely with Palliative Care Units. The setting up of Rumah Hospis in Penang would enhance palliative care in Penang. Home care nursing by some government general hospitals serves to enhance the continuity of care of terminally ill patients after discharge from hospital. For example, Hospital Kuala Lumpur started such service in 1995 for patients within a 10-kilometre radius of the hospital.

In East Malaysia, Palliative Care Programmes have been started in Palliative Care Units as well as Hospice Home Care as a part of a community-based service. The programmes include the training of doctors, nurses, medical assistants and lay volunteers in the palliative care of terminally ill cancer patients. Palliative Care Units have been set up in several government hospitals, such as Queen Elizabeth Hospital in Kota Kinabalu in 1995, and they have untaken training of Ministry of Health personnel. There are 11 Palliative Care Units and 48 Palliative Care Teams in various hospitals in the Ministry of Health, and 17 hospice organisations (NGOs) under an umbrella organisation called the National Hospice Council that was formed in June 1998.

The Palliative Care Unit of Hospital Selayang was started in December 2002 as part of the Ministry of Health's policy to support palliative care services in all government hospitals in Malaysia. As Hospital Kuala Lumpur was largely overcrowded and there was no space for the development of a new ward, it was decided that the new Palliative Care Unit be started in Hospital Selayang, which is also a regional centre for pain control.

## TRAINING

Training is an integral part of any cancer programme. The number of specialists in cancer treatment and the facilities available need to be increased to cope with the workload. There are 39 clinical oncologists in Malaysia of whom 23 are in the private sector. Compared to the United Kingdom which has a ratio of 8 oncologists per million population [23], Malaysia now has 1.6 per million population. Clearly, one of the main immediate concerns is to address the acute shortage of oncologists. Physicists number 26, while there are 104 therapy radiographers, 2 medical technologists and 45 nurses who have undergone post-basic oncology training.

**Figure 10:**
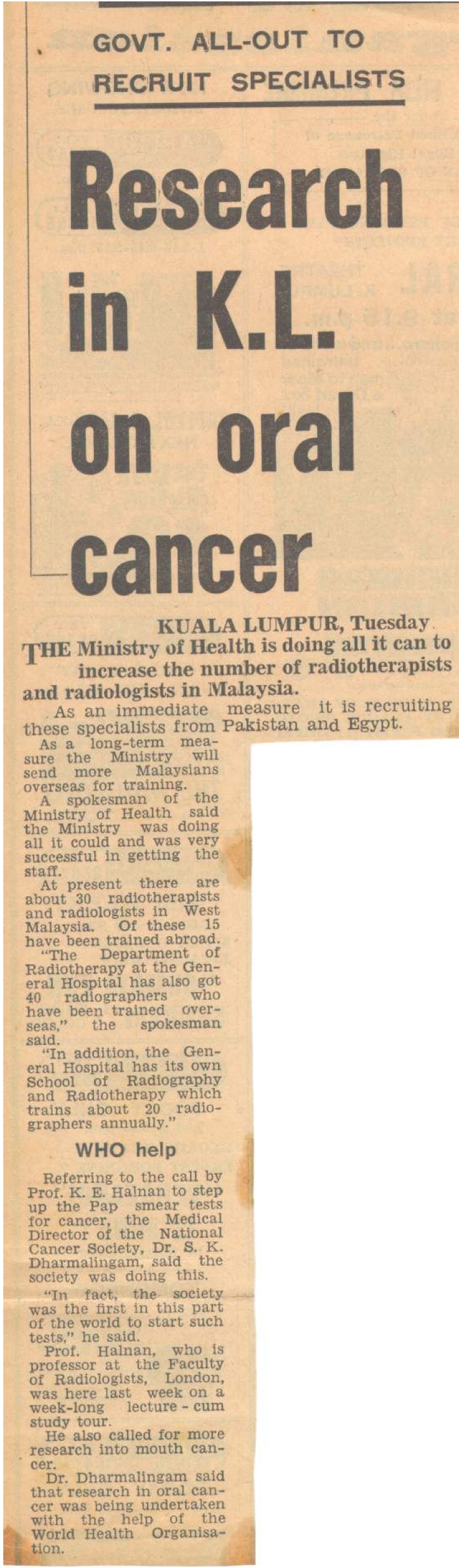
Efforts to recruit foreign specialists in the 1970's.

In 1971, it was reported that there were about 30 radiotherapists and radiologists in West Malaysia, and the Ministry of Health at that time was doing all it could to increase the number of these specialists (Figure 10). The measure adopted then was recruitment from Pakistan and Egypt, while the long term plan was to send more Malaysians overseas for training [16].

The School of Radiography was started at the General Hospital Kuala Lumpur in 1963. Mr. A.R. Hutchinson was the Radiotherapy Advisor [1]. The first five student radiotherapy radiographers commenced training in October 1967 and sat for the examination in 1969.

In 1981 the Ministry of Health Malaysia curriculum was introduced and the training period was increased to 3 years. On 6 July 1995 the name for School of Radiography and Radiotherapy was changed to College of Radiography and Radiotherapy, Kuala Lumpur Hospital. From January 2002, the administration of the College of Radiography and Radiotherapy was transferred from Kuala Lumpur Hospital to the Department of Health, Federal Territory Kuala Lumpur [17]. The post basic course for therapy radiographers in Radiation Therapy Planning was started in August 1998 at the College of Radiography and Radiotherapy, Kuala Lumpur. The College of Radiography and Radiotherapy was officially shifted from the Malaysian Medical Association Building in Jalan Pahang to Sg. Buloh on 31 December 2004 [18].

The post basic course for Oncology Nursing was started at the School of Nursing in Kuala Lumpur on 1 October 1996.

The First ASEAN Association of Radiologists' Scientific Meeting was held from 30 October to 1 November 1980 at the Faculty of Medicine, University of Malaya. Invited speakers included Professor J.G. Bloom from the Royal Marsden Hospital, London.

The International Atomic Energy Agency organised the IAEA Regional Training Course on Brachytherapy of uterine cancer using Manual and Remote After-loading Techniques at the Faculty of Medicine, National University Hospital and General Hospital of Kuala Lumpur from 6-26 October 1986. It was organised in cooperation with the Government of Malaysia and Universiti Kebangsaan Malaysia, in collaboration with General Hospital Kuala Lumpur and the Nuclear Energy Unit of the Prime Minister's Department. The Organising Committee comprised Professor I. Saad, Dr. P. Singh, Dr. M.T. Azhar, Dr. A. Lim, Mr. J.T. Wong, and Mr. T. Yogaratnam. Lecturers included Mr. H. Tsujii, Dr. S. Ganesan and Dr. M.K. Tan. Participants comprised 23 representatives from 10 countries and one representative from the Middle East. The Ralstron B 20 (Shimadzu Corp., Kyoto, Japan) that was generously donated by the Government of Japan and channelled through the IAEA to Malaysia, was an essential component of the training course [19], and was the first remote afterloading brachytherapy machine installed in Malaysia.

Regional cancer meetings in which Malaysia has taken part included Asian Oceanian Congress of Radiology (June 1995) and Asia Pacific Cancer Congress (October 1996) [19]. The First Malaysia/IAEA Brachytherapy Course was jointly organised by the Ministry of Health Malaysia and Hospital Universiti Kebangsaan Malaysia from 24-27 February 2004, with cooperation from the Malaysian Institute of Nuclear Technology and Research (MINT) and was attended by 93 participants from around the world. The foreign expert was Professor S. Nag. The participation and cooperation between the government, university and private sector was remarkable.

While post-graduate training in radiotherapy and oncology had traditionally been in the United Kingdom and Ireland, the Malaysian Government sent two trainees to Hong Kong for the first time in 2002. This was due to the increasing difficulties encountered by our trainees in pursuing post-graduate training in the United Kingdom. The Master of Clinical Oncology was started in University of Malaya in November 2002, with strong support from the Ministry of Health. The first professional examination was held in November 2003, with Professor M. Barton and Professor Dato' Dr. M.T. Azhar as external examiners from Sydney and International Islamic University, respectively. The external examiners gave favourable and encouraging reports regarding the standards and conduct of the examination.

As evidenced by the First Report of the National Cancer Registry of Malaysia [10] and Second Report of the National Cancer Registry of Malaysia [21], it cannot be overemphasised that the optimal number of oncologists needed must be much higher than the number available at the moment. Both overseas training and a local post-graduate course for clinical oncology are being supported simultaneously by the government to train more clinical oncologists as quickly as possible.

## CONCLUSIONS

Good and comprehensive cancer treatment is the right of all. Efforts are being intensified to improve cancer services in the country, with emphasis on training of skilled manpower, equipment and facilities. The blueprint includes the setting up of the National Cancer Institute, the strengthening of existing centres as well as establishing new facilities. Networking between the various sectors will be given more emphasis. Multidisciplinary care will be further strengthened.

The Ninth Malaysia Plan (2006-2010) will see a concerted effort to improve the various aspects of cancer control which includes prevention of cancer, developing and enhancing programmes for diagnosis and early detection, treatment, rehabilitation and palliative care. The role of herbal, traditional and complementary therapies will be explored.

While there is a great need to build facilities and train more staff skilled in cancer management, it is equally vital to ensure that cancer care services are accessible and affordable throughout the entire health system, from primary care level up to the centres for tertiary care, throughout the whole country.
